# Voltage-Gated Ca^2+^-Channel α1-Subunit *de novo* Missense Mutations: Gain or Loss of Function – Implications for Potential Therapies

**DOI:** 10.3389/fnsyn.2021.634760

**Published:** 2021-03-03

**Authors:** Jörg Striessnig

**Affiliations:** Department of Pharmacology and Toxicology, Institute of Pharmacy, Center for Molecular Biosciences Innsbruck, University of Innsbruck, Innsbruck, Austria

**Keywords:** calcium-channels, channelopathies, mutations, neurodevelopmental disorders, gating pore currents

## Abstract

This review summarizes our current knowledge of human disease-relevant genetic variants within the family of voltage gated Ca^2+^ channels. Ca^2+^ channelopathies cover a wide spectrum of diseases including epilepsies, autism spectrum disorders, intellectual disabilities, developmental delay, cerebellar ataxias and degeneration, severe cardiac arrhythmias, sudden cardiac death, eye disease and endocrine disorders such as congential hyperinsulinism and hyperaldosteronism. A special focus will be on the rapidly increasing number of *de novo* missense mutations identified in the pore-forming α1-subunits with next generation sequencing studies of well-defined patient cohorts. In contrast to likely gene disrupting mutations these can not only cause a channel loss-of-function but can also induce typical functional changes permitting enhanced channel activity and Ca^2+^ signaling. Such gain-of-function mutations could represent therapeutic targets for mutation-specific therapy of Ca^2+^-channelopathies with existing or novel Ca^2+^-channel inhibitors. Moreover, many pathogenic mutations affect positive charges in the voltage sensors with the potential to form gating-pore currents through voltage sensors. If confirmed in functional studies, specific blockers of gating-pore currents could also be of therapeutic interest.

## Introduction

Decreasing cost for next-generation sequencing (NGS) has allowed to diagnose pathogenic mutations in an increasing number of patients affected by genetic diseases. Genome-wide association studies mainly identify disease-associated common genetic variants, which explain only a very small proportion of overall phenotypic variance. In contrast, trio-based whole-exome or whole-genome sequencing genetic studies in well-phenotyped patient cohorts can detect rare, recurrent damaging variants of large effect size that can account for most phenotypic variance and can be considered causative (Sullivan and Geschwind, 2019). Such variants explain a wide variety of symptoms, especially in early-onset sporadic pediatric genetic disorders (Consortium et al., 2013; Allen et al., 2017; Satterstrom et al., 2020). This informs not only diagnosis and genetic counseling, but can also refine a patients’ prognosis, initiate surveillance for other symptoms in multisystem disorders and guide therapeutic decision-making (Helbig et al., 2016; Weber et al., 2017; Noebels, 2019). In addition, the identification of multiple risk genes within defined signaling networks provides valuable novel insight into dysregulated molecular pathways underlying a pathology.

Disorders that have strongly benefited from NGS – based genetic diagnostics are neuropsychiatric and neurodevelopmental disorders, including epilepsies, in particular childhood epilepsies, autism spectrum disorders (ASD), and intellectual disability (ID) with a large collective contribution of rare, large-effect pathogenic variants (Allen et al., 2017; Howard and Baraban, 2017; Weber et al., 2017; Myers et al., 2020; Satterstrom et al., 2020). Rare large-effect variants in the same gene may not be specific for one of these disorders (e.g., ASD, Myers et al., 2020) and other sources of genetic variation, such as polygenic risk from common variants or genetic mosaicism, as well as environmental factors can affect phenotypic expression. Whereas the significance of a novel *de novo* mutation identified only in a single individual is difficult to interpret, the identification of a *recurrent* pathogenic variant strongly supports a large genetic contribution to the phenotype in an individual within this disease spectrum. In some cases, this can even guide therapies either with new drugs or by repurposing drugs already licensed for other indications. Although often experimental in nature and mainly symptomatic, such individualized therapies can enhance the quality of life for affected individuals and their caregivers. For example, everolimus has been repurposed for the treatment of Tuberosclerosis TSC1 and TSC2 (Krueger et al., 2016; Samueli et al., 2016), and 5-hydroxytryptophan is used to treat patients with DOPA-responsive dystonia (DRD, OMIM #128230) caused by Sepiapterin Reductase Deficiency (Bainbridge et al., 2011). Among patients with epilepsy referred to diagnostic whole exome sequencing the diagnostic yield of characterized disease genes is > 30% (Helbig et al., 2016; Symonds and McTague, 2020) and genetic diagnosis has significant impact on patient management, such as choosing or avoiding certain antiepileptic medications (Symonds and McTague, 2020).

Some rare disease phenotypes are associated with only a single or a few defective genes (like TSC or DRD). More common, pathophysiologically more complex, and clinically often overlapping phenotypes such as ASD, childhood epilepsies, ID or cardiac arrhythmias are associated with a larger number of risk genes (e.g., over 100 in ASD and more than 650 in ID; Kochinke et al., 2016; Satterstrom et al., 2020). However, most of them account only for a very small fraction of affected individuals. This increases the probability that a new sporadic variant of unknown pathogenicity is found in one of these genes. In the case of *de novo* mutations (DNMs, newly formed during gamete development or very early in embryonic development, not inherited from the parents), bioinformatics pipelines can help to separate likely disease - causing from other variants. This involves eliminating variants exceeding a defined allele frequency in likely unaffected control populations as, for example, available in the gnomAD database (Karczewski et al., 2020). Validated DNMs can then be classified according to the predicted impact of the genetic variation on gene and protein function. A protein loss of function (pLOF) can reliably be predicted from *de novo* deleterious, likely gene-disrupting mutations, such as nonsense, stop gain, splice site or frameshift variants. In contrast, *missense* DNMs change the amino acid composition of a protein but usually do not prevent its complete translation. Therefore, their functional consequences are more difficult to predict: they can be functionally silent, induce a decrease (**loss-of-function, LOF**) or an increase (**gain-of-function, GOF**) of protein function. On average each offspring harbors at least one *de novo* protein – coding missense variant (Gratten et al., 2013; Krumm et al., 2015). Therefore, in diseases with many risk genes (see above) the probability of observing a new missense DNM in one of them by chance is high but its pathogenicity remains often unclear, even if present in a known high risk gene. Therefore, standards and guidelines for the interpretation of sequence variants have been developed by the American College of Medical Genetics (ACMG, Richards et al., 2015) to classify variants according to their predicted pathogenicity (“pathogenic”, “likely pathogenic”, “uncertain significance”, “likely benign”, and “benign”). Ideally, functional assays are available to test for GOF or LOF changes, which can support classification as is the case for voltage-gated Ca^2+^-channel (Cavs, see section “Functional Analysis of Protein-Coding Missense Variants”).

This review summarizes our current knowledge of disease-relevant genetic variants within the family of voltage gated Ca^2+^-channels. A special focus will be on missense DNMs in their pore-forming α1-subunits, which are increasingly reported in NGS studies and can help to explain sporadic cases of Ca^2+^-channel-associated pathologies. These include epilepsies, ASD, ID, developmental delay, cerebellar ataxias, severe cardiac arrhythmias, sudden cardiac death and endocrine disorders such as congential hyperinsulinism and hyperaldosteronism. Since potential personalized therapeutic approaches depend on whether a missense DNM induces a loss- or gain of channel function, we will discuss the complex question of how altered gating of these ion channels can translate into such functional changes.

First, this review provides a brief overview about the ten members of the Cav-channel family and highlights the most common macroscopic gating changes induced by single amino acid substitutions. Finally, the spectrum of variants known to cause Ca^2+^-channelopathies is described. Since the protein structures of the pore-forming Ca^2+^-channel α1-subunits are highly conserved (Wu et al., 2016; Zhao et al., 2019a, b; Catterall et al., 2020), we will also discuss if the location and functional effects in one of the Cav family members can help to predict pathogenicity of a novel variant in another Cav. This could refine predictions based on the ACMG guidelines and thus help in genetic counseling.

## The Voltage-Gated Ca^2+^-Channel Family

Excellent reviews have recently been published on this topic (Zamponi et al., 2015; Catterall et al., 2020; Dolphin and Lee, 2020; Ablinger et al., 2020) and valuable detailed information on the physiology, tissue distribution and pharmacology of these channels can be found in the NC-IUPHAR Guide to Pharmacology^1^ (Alexander et al., 2019).

**FIGURE 1:**
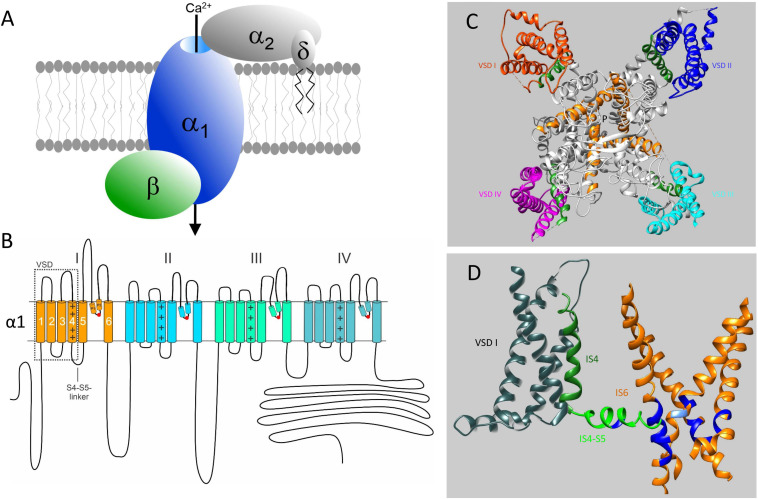
Subunit structure of voltage-gated Ca^2+^ channels and their mutation-sensitive regions. **(A)** The pore-forming α1-subunit determines most of the biophysical properties of voltage-gated Ca^2+^ channels and also carries the binding domains for subtype-selective drugs and toxins. β-and α2δ-subunits associate with α1-subunits of Cav1 and Cav2 channels but not with Cav3 T-type channels. They support channel trafficking to the membrane, fine-tune gating properties but appear to have also channel-independent functions. Here we only discuss pathogenic mutations in α1-subunit genes. **(B)** Transmembrane folding topology of α1-subunits. Four homologous domains (I-IV) each form a voltage sensing domain (VSD, S1-S4; dotted box). The S6 helices together with the connecting S5-S6 linkers of each domain contribute to the formation of a single central Ca^2+^-selective pore. The S4-S5 linkers in each domain transmit the voltage-dependent conformational changes of the S4-helix movements to the cytoplasmic side of the pore by interactions with adjacent S4-S5 – linkers (Hofer et al., 2020) and S6 helices (Wu et al., 2016; Catterall et al., 2020). Mutations neutralizing the voltage-sensing positive charges in each voltage sensor can open an additional ion conducting pathway (termed ω-pore) through the voltage –sensing domain, which can conduct pathogenic gating pore currents (see Figure 5 and text for details). P1 and P2 are helices contributing to the formation of the external part of the pore. They coordinate the formation of the Ca^2+^-selectivity filter formed by four negatively charged amino acid residues indicated by the red circles. **(C)** Cryo-electron microscopy structure of the Cav1.1 calcium channel complex purified from rabbit skeletal muscle (PDB 5GJV). Only a top view of the pore-forming α1-subunit is shown to illustrate the position of the four voltage-sensing domains (VSD I – IV, highlighted in different colors). The voltage-sensing positively charged S4 helices of the VSD domains are shown in green (positive charges are not indicated). The central pore-forming region including the intracellular activation gate are formed by the S6 helices indicated in orange. P indicates the ion conducting pathway. The structure is in a presumably inactivated state with S4 voltage-sensors “up” and the activation gate closed (Wu et al., 2016). **(D)** Most of the missense mutations (in particular GOF mutations) causing Ca^2+^-channelopathies occur in regions important for voltage-dependent channel gating. These functional modules consists of S4 (green) and the cytoplasmic S4-S5 linkers (light green), which are tightly coupled through multiple interactions (not illustrated) to the activation gate formed by the four S6 helices (orange). For clarity the VSD (gray), S4 and S4-S5 linker are only shown for domain I together with all 4 S6 helices (orange). Positions where pathogenic mutations occur in all Cavs (Figures 3, 4) are indicated in blue. In IS4-S5 this represents the position of the FHM1 mutation S218L (*CACNA1A*) and the CSNB2 mutation S229P (*CACNA1F*), which both cause GOF (type 2 gating; see text for details). The position of the Timothy Syndrome mutation G402R/S (*CACNA1C*) is indicated in light blue. Note that pathogenic GOF mutations at the same position also occur in Cav1.3, Cav1.4, and Cav2.3 (Figure 3). The schemes in **(C,D)** were generated using UCSF Chimera 1.13.1 (Pettersen et al., 2004). The position of mutations in the Cav1.1 α1-subunit is shown based on the sequence alignments in Figures 3, 4 and does not account for potential differences in the folding structure of the different α1-subunits.

Cavs are found in all electrically excitable cells. Voltage-dependent Ca^2+^ influx through Cavs serves a dual function in cell signaling. It generates intracellular Ca^2+^ signals controlling Ca^2+^-dependent cellular processes. In addition, Ca^2+^ ion inward current also drives membrane depolarization and thus directly contributes to a cell’s electrical activity (Zamponi et al., 2015). Consequently, Cavs are essential for key physiological functions, such as learning and memory, neurotransmitter and hormone release, muscle contraction, cardiac pacemaking and sensory functions, including hearing and visual function (Zamponi et al., 2015; Nanou and Catterall, 2018; Pangrsic et al., 2018). Ten types of Cavs are formed by different pore-forming α1-subunits (Figure 1), each encoded by a separate gene (Zamponi et al., 2015; Alexander et al., 2019) (Table 1). Differences in their biophysical properties, tissue expression, subcellular targeting, association with other interacting proteins in signalosomes and in their modulation by other signaling pathways generates the functional diversity required for the many physiological functions they support (Zamponi et al., 2015; Nanou and Catterall, 2018; Liu et al., 2020). Extensive alternative splicing and association with various modulatory accessory subunits (β1-β4, α2δ1-α2δ4), posttranslational modifications and RNA-editing (Huang et al., 2012; Zamponi et al., 2015; Li et al., 2017; Loh et al., 2020) further fine-tunes their biophysical and pharmacological properties (Buraei and Yang, 2010; Zamponi et al., 2015; Ablinger et al., 2020). This requirement for tight functional control explains why even minor changes in the activity of these channels induced by genetic variants can cause human disease.

**TABLE 1:** Voltage-gated Ca^2+^ channels: classification and human genetic diseases.

Family	α 1-Subunit Gene	α 1-Subunit	Channelopathies (OMIM)
Cav1 (L-type)	*CACNA1S*	Cav1.1	• Hypokalemic Periodic Paralysis Type 1 (# 170400, AD, DN) • Normokalemic Periodic Paralysis (#170600, AD) • Malignant Hypothermia Susceptibility 5 (#601887, AD)
	*CACNA1C*	Cav1.2	• Timothy Syndrome (#601005, AD) • Long QT Syndrome 8 (LQT8) (#618447, AD) • Brugada Syndrome 3 (#611875)
	*CACNA1D*	Cav1.3	• Sinoatrial Node Dysfunction and Deafness Syndrome (#614896, AR)[ • Primary aldosteronism, seizures, and neurologic abnormalities (#615474, DN) • Autism spectrum disorder (with or without more severe manifestations including intellectual disability, neurological abnormalities, primary aldosteronism and/or congenital hyperinsulinism (not listed in OMIM, DN) • Aldosterone producing adenomas (APAs, somatic, DN)
	*CACNA1F*	Cav1.4	• Congenital Stationary Night Blindness Type 2 (#300071, XL) • X-linked Cone-Rod Dystrophy 3 (#300476, XL) • Aland Island Eye Disease (#300600, XL)
Cav2	*CACNA1A*	Cav2.1 (P/Q-type)	• Familial and Sporadic Hemiplegic Migraine Type 1 with or without progressive cerebellar ataxia (#141500, AD, DN) • Episodic Ataxia Type 2 (#108500, AD) • Spinocerebellar Ataxia Type 6 (#183086, AD)* • Early Infantile Epileptic Encephalopathy 42 (#617106, AD) • Congenital Ataxia*
	*CACNA1B*	Cav2.2	• Neurodevelopmental disorder with seizures and non-epileptic hyperkinetic movements (#618497, AR)
	*CACNA1E*	Cav2.3	• Early Infantile Epileptic Encephalopathy 69 (#618285, AD)
Cav3 (T-type)	*CACNA1G*	Cav3.1	• Spinocerebellar Ataxia Type 42 (#616795, AD) • Spinocerebellar Ataxia Type 42 early-onset, with neurodevelopmental deficits (Childhood-Onset Cerebellar Atrophy; #618087, AD)
	*CACNA1H*	Cav3.2	• Familial Hyperaldosteronism type IV (#617027, AD) • Aldosterone producing adenomas (APAs, somatic, DN)
	*CACNA1I*	Cav3.3	• Neurodevelopmental disorder with epilepsy and intellectual disability (not yet listed in OMIM)

*Human diseases caused by inherited and *de novo* missense Ca^2+^ channel α1-subunit variants. Only diseases are listed in which the high pathogenic potential of mutations is either evident from their high penetrance in families or from functional studies of recurrent mutations in *de novo* variants. ^∗^, phenotypic and genotypic overlap with other channelopathies of this gene. AD, inheritance: autosomal dominant; AR, autosomal recessive; XL, X-linked; DN, *de novo*. For most familial disorders with autosomal dominant inheritance pathogenic *de novo* mutations have also been described.*

Based on sequence homology between their pore-forming α1-subunits (Figure 1) as well as their functional and pharmacological properties, three Cav families are distinguished: Cav1, Cav2 and Cav3 (Table 1; Alexander et al., 2019).

Cav1.1-Cav1.4 α1- subunits form the family of **L-type** Ca^2+^-channels (Cav1). They have unique sensitivity to low nanomolar concentrations of dihydropyridine (DHP) Ca^2+^-channel blockers (Alexander et al., 2019; Table 1). Cav1.2 and Cav1.3 are expressed, often together, in most electrically excitable cells (Zamponi et al., 2015). In contrast, Cav1.1 and Cav1.4 expression and function is largely restricted to skeletal muscle (Cav1.1) and the retina (Cav1.4). Cav1.2 controls cardiac inotropy and arterial vascular tone (Zamponi et al., 2015). It is the main therapeutic target for Ca^2+^-channel blockers (such as amlodipine, felodipine), which are approved since decades for the treatment of angina and hypertension (Striessnig and Ortner, 2020). Cav1.3 activates at more negative voltages than Cav1.2 (Koschak et al., 2001; Zamponi et al., 2015). Therefore it serves as a pacemaker channel in the sinoatrial and AV-node (Marger et al., 2014) and is essential for cochlear inner hair cell function and hearing (Zamponi et al., 2015). Cav1.2 and Cav1.3 are both present in central neurons, predominantly postsynaptically at dendritic spines. Both channels control the short- and long-term regulation of neuronal activity in several brain circuits, and contribute to different types of learning, memory and emotional behaviors (Zamponi et al., 2015; Kabir et al., 2017; Nanou and Catterall, 2018).

Members of the Cav2 family (Cav2.1-Cav2.3, giving rise to **P/Q-, N- and R-type** voltage-gated Ca^2+^ currents) are also located at pre-synaptic active zones and support fast neurotransmitter release in neurons. Together with L-type channels they also trigger hormone release in endocrine cells (Zamponi et al., 2015).

Low-voltage activated Cav3 channels (Cav3.1-Cav3.3) comprise the family of **T-type** Ca^2+^-channels. They activate and inactivate at more negative membrane potentials than Cav1 (including Cav1.3) and Cav2 (Perez-Reyes and Lory, 2006; Zamponi et al., 2015). This negative operation range allows them to be active at subthreshold voltages, and provides them with a prominent role for integration and control of neuronal firing patterns (Kim et al., 2001; Zamponi et al., 2015).

Recently, the cryo-EM structures of the pore-forming α1-subunits of two members of the Cav family have been solved in complex with selective channel blockers: Cav1.1 (Wu et al., 2016; Zhao et al., 2019b; Gao and Yan, 2020) and the evolutionary more distantly related Cav3.1 (Zhao et al., 2019a) channel. Together with previous work on related channels (Catterall et al., 2020), these structures provide exciting new information about the molecular details involved in voltage-sensing, channel gating (Figures 1C,D, based on the cryo-EM structure of Cav1.1; Wu et al., 2016) and ion permeation as well as isoform-specific functional and pharmacological differences. As will be discussed below, these common structural features can be instrumental for predicting functional consequences of disease-causing mutations across different α1-subunits.

## Gain- and Loss- of Ca^2+^-Channel Function Induced by Missense Mutations

For proper function and subcellular targeting Cav1 and Cav2 channels require association with one of four different β- and one of four different α2δ-subunits (Figure 1A; Buraei and Yang, 2010; Zamponi et al., 2015; Ablinger et al., 2020). Therefore, genetic variants in any of these accessory subunits can indirectly affect channel function. Signaling changes resulting from genetic variation in these subunits are mechanistically more complex to interpret, because any of the four different β- and of the four different α2δ isoforms may associate with the different Cav1 and Cav2 α1- subunits in a given cell (such as a neuron). Moreover, these subunits also appear to serve functions independent of Cavs (Hofmann et al., 2015; Dolphin, 2018; Striessnig, 2018). This review therefore exclusively focuses on channelopathies resulting from genetic variants in the pore forming α1-subunit genes. These can be directly related to the known physiological functions of a particular channel type and thus provide more specific hints for molecular disease mechanisms.

### Functional Analysis of Protein-Coding Missense Variants

As mentioned above, the functional consequences of protein-coding missense DNMs (including in-frame deletions/insertions) are more difficult to predict and different amino acid substitutions at the same position may even cause opposite functional effects (Hofer et al., 2020). While missense DNMs may also induce a LOF by generating functionally silent channels (e.g., by an in-frame glycine insertion in Cav1.3 α1; Baig et al., 2011), they can have more complex effects by affecting channel gating or ion conductance. In this case, it is difficult to predict if a given gating change enhances or reduces channel activity in the context of a cell’s characteristic firing pattern. It is very important that we keep this in mind when referring to a “GOF” phenotype. It only implies that functional changes induced by a mutation can *permit* enhanced channel activity and Ca^2+^ influx *under certain circumstances*, such as during a given electrical firing pattern of a cell. As outlined below, it does not necessarily exclude that the same “GOF” variant can also *reduce* channel activity in a different functional context.

Analysis of mutation-induced functional changes in cells expressing Cavs is complicated by the fact that tissues cannot be routinely obtained from patients (skeletal muscle biopsies are an exception). Moreover, individual Cavs are difficult to study in native tissues, because often currents of many different Cav types contribute to the total Ca^2+^ current in a cell. This requires isolation of the current component of interest, which is possible for some (e.g., Cav2.1-mediated P/Q-type currents in cerebellar Purkinje cells, Cav1.2-mediated L-type currents in cardiomyocytes; see below) but very difficult for others (e.g., Cav1.3-mediated L-type currents, Cav2.3-mediated R-type currents; Forti and Pietrobon, 1993; Sinnegger-Brauns et al., 2004). Moreover, currents recorded in native cells expressing heterozygous variants are “contaminated” by wildtype currents, making subtle changes of mutant current components more difficult to detect than in a heterologous system (Miki et al., 2008).

These restrictions require expression of missense variants in mammalian heterologous systems, such as HEK-293 or baby hamster kidney cells, and quantification of functional changes in patch-clamp experiments. In general, it appears that macroscopic gating changes observed in heterologous expression systems reproduce the changes observed in native cells reasonably well. This is evident from animal models harboring specific human mutations (Drum et al., 2014; Rose et al., 2014; Calorio et al., 2019) or from human cells differentiated from patient-derived induced pluripotent stem cells (iPSCs; e.g., neuron-like or cardiomyocyte-like cells; Yazawa et al., 2011; Krey et al., 2013; Song et al., 2017; Estes et al., 2019; Chavali et al., 2019). In contrast, it is less clear how well changes of current density and of channel protein expression observed in heterologous overexpression systems reflect changes of current density in native tissues (Chavali et al., 2019). Obviously, many other regulatory factors absent in heterologous expression systems, in particular cell-type specific protein-protein interactions, affect channel protein stability and its plasma membrane-targeting in their native environment.

Special attention should also be paid to missense mutations located in alternatively spliced exons, which may restrict the mutation only to channels expressing this exon. Conversely, a single missense mutation may even promote the expression of the exon in which it is located. This has recently been shown in iPSC-derived neural progenitor cells and neurons (Panagiotakos et al., 2019) generated from patients with Timothy syndrome (see below). The GOF mutation G406R in exon 8a of *CACNA1C* (Cav1.2α1; see section on *CACNA1C* below) inhibits the developmental splicing switch to exon 8 – containing Cav1.2 channels, which do not harbor the mutation. This favors extended use of the mutant, exon 8a-containing channel permitting enhanced mutant channel activity during brain development. This is likely due to the position of the mutation in the splice acceptor site of exon 8a (Panagiotakos et al., 2019).

### Channel Loss of Function Mutations

Like for other proteins (see above), genetic variants can cause disease (or increase disease risk) by disrupting channel function. This can be predicted for likely gene-disrupting mutations expected to prevent the synthesis of functional α1-subunit proteins (pLOF). If no protein is made from the defective allele, heterozygous LOF mutations may cause disease resulting from *haploinsufficiency*. However, if a defective (e.g., truncated) protein is still synthesized it may even exert a *dominant negative* effect on the wildtype channel produced from the unaffected allele. This requires a mechanism enabling functional coupling between (wildtype and mutant) α1-subunits. In addition, the mutant RNA must escape nonsense-mediated RNA decay (Holbrook et al., 2004). In the case of Cavs such dominant negative effects appear likely: substantial expression of mRNA transcripts from mutated alleles of both frameshift and nonsense mutations (in the case of Cav2.1) has been reported in humans (Sintas et al., 2017; Balck et al., 2018). Moreover, binding of misfolded mutants to wildtype α1-subunits occurs *in vitro* thereby promoting channel degradation (Mezghrani et al., 2008; Page et al., 2010). It is therefore possible that some heterozygous LOF mutations will reduce channel activity even below 50%, a consideration especially important for understanding the pathology of Cav2.1 channelopathies (see below). However, it should be kept in mind that the *in vitro* studies were carried out with cDNA constructs in heterologous overexpression systems with yet unclear relevance for the *in vivo* situation, and the detection of transcripts of the mutant alleles is still very indirect evidence.

### Common Macroscopic Gating Changes Induced by Protein-Coding Missense Mutations

**FIGURE 2:**
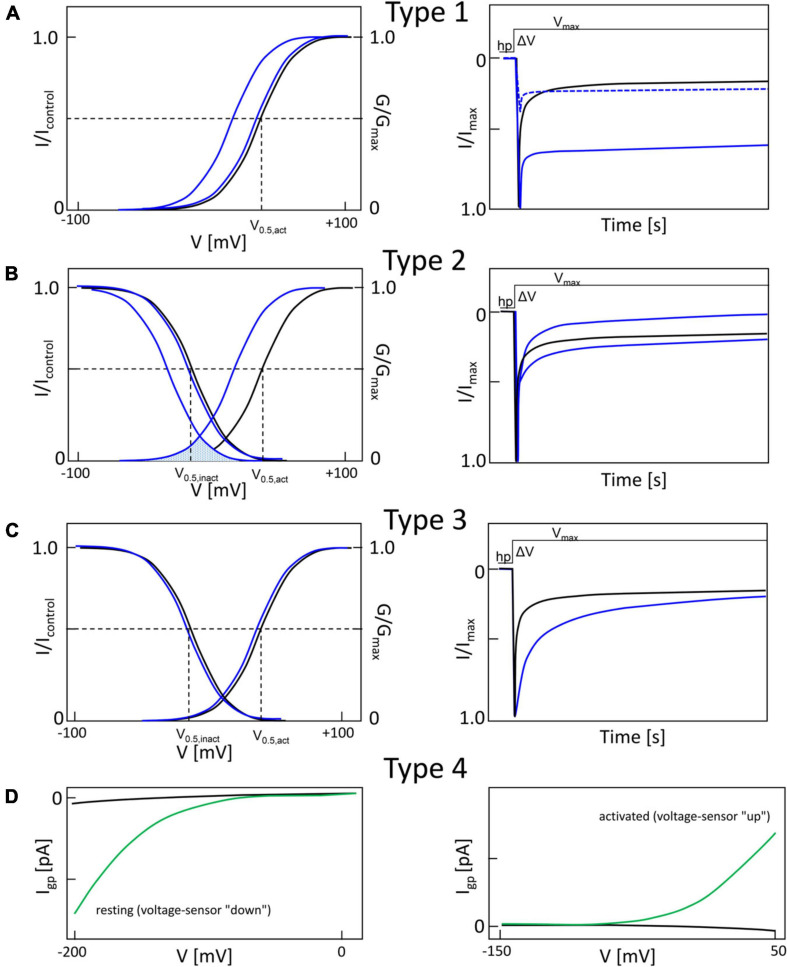
Typical gain-of-function macroscopic gating changes described for inherited or *de novo* missense mutations in voltage-gated Ca^2+^ channel α1-subunits. To facilitate discussion the most frequently observed gating changes are classified into types 1-4. For details see text. Mutant current properties are indicated in blue **(A–C)** or green curves **(D)**, wildtype current in black. **(A)** Type 1 is characterized by the appearance of a large fraction of a non-inactivating current component (as e.g., during long depolarizations from a negative holding potential to the potential of maximal inward current; right panel). Changes in the voltage-dependence of activation gating (left) may or may not be present (blue curves, left). Note that even if maximal inward current is reduced by a mutation, current amplitude may exceed wildtype current later during depolarization due to the slowly inactivating current component (right, dotted curve). **(B)** The main feature of type 2 changes is a strong shift of the voltage-dependence of activation to more negative voltages independent of smaller effects on current inactivation during depolarizations. The voltage-dependence of inactivation may or may not be shifted to more negative voltages (left). The voltage range in which steady-state inward current (“window current”) may be observed (i.e., the overlap of voltage-dependence of activation and inactivation curves) is indicated for mutant current (shaded) **(C)**. Some mutations do not affect the voltage-dependence of gating (left) but cause a slowing of inactivation (weaker than in type 1). **(D)** As described in Figure 5, gating pore currents are enabled by mutations of an S4 gating charge (green lines). The position of the mutation relative to the hydrophobic constriction site (HCS, Figure 5) determines if the pore is open during the hyperpolarized “down”-state of the S4 helix at negative voltages (left) or during the depolarized “up”-state at positive voltages (right). In the “down” state, at potentials near the K^+^-equilibrium potential, inward gating-pore currents would be primarily carried by Na^+^. In the “up” state, at potentials positive to the activation threshold of the channel, potassium outward gating-pore current predominates. However, upon fast repolarization of the action potential to negative potentials Na^+^ inward gating-pore current may predominate until the voltage sensor moves back to the “down” position” (especially relevant in “slowly inactivated” Na^+^-channels,” for details see ref. Sokolov et al., 2008). G/G_max_, normalized conductance (steady-state activation); I/I_max_, normalized inward current (steady-state inactivation). V_0_._5,act_, V_0_._5,inact_, half maximal voltages of activation and inactivation; hp, holding potential; ΔV, depolarization from a negative holding potential to the voltage of maximal inward current (V_max_).

**FIGURE 3:**
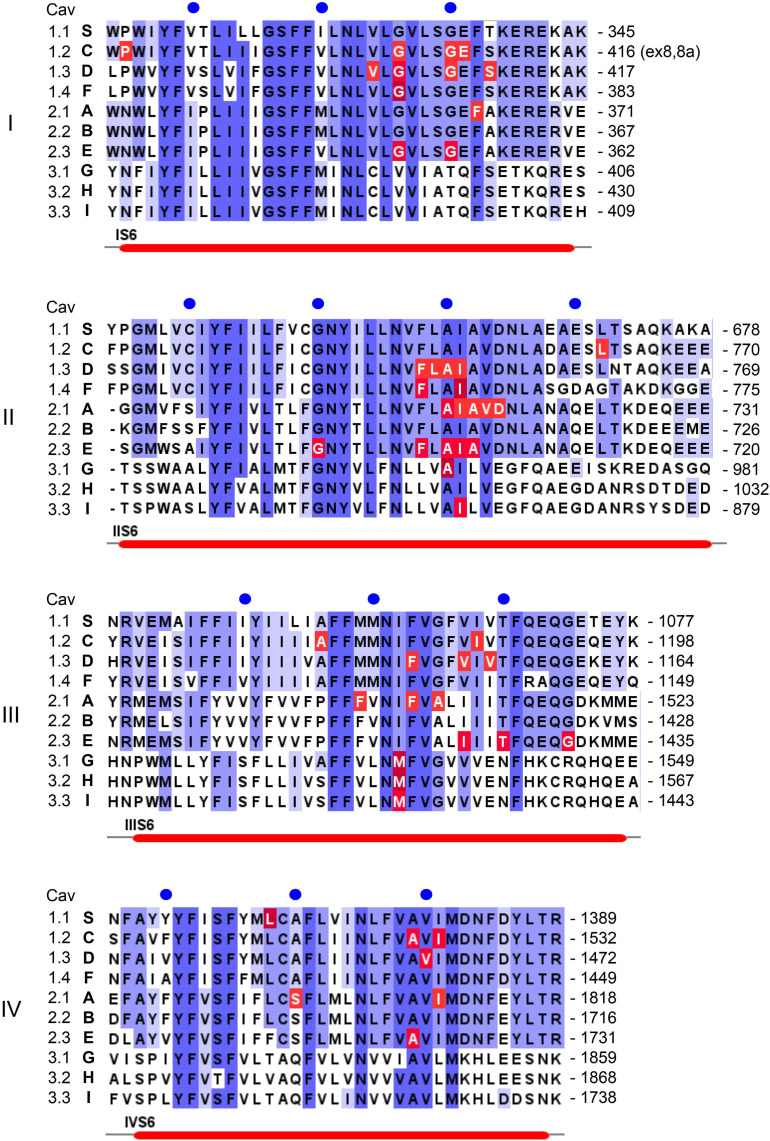
Position of pathogenic mutations within S6 helices of the ten different Ca^2+^ channel α1-subunits. Sequence alignment and labeling is as described in legend to Figure 4.

Missense-mutations can affect the macroscopic gating properties of Cavs in a complex manner, evident as changes of the voltage-dependence of current activation and inactivation. These alterations are very similar across different Cavs. For further discussion Figure 2 illustrates the most frequently observed gating changes in a simplified manner as type 1 – 4, although overlaps exist between them (Pinggera et al., 2018; Ortner and Striessnig, 2020). A gating change unambiguously interpretable as a phenotype permitting enhanced channel function (GOF) is illustrated in panel A (“type 1”). Its hallmark is an almost complete failure of a large fraction of current to inactivate during depolarization. This is often quantified in whole-cell patch-clamp experiments during square pulse depolarizations (ΔV) from a negative holding potential (hp) to the maximum of the voltage of maximal inward current (V_max_, Figure 3A, right panel). This failure to inactivate can even prevent meaningful measurements of steady-state inactivation. At the same time, the half-maximal activation voltage (V_0_,_5,act_) can be either unchanged or shifted to more negative voltages (blue curves, Figure 2A, left panel). Note that the time course of inactivation is controlled by Ca^2+^-dependent as well as voltage-dependent inactivation. The latter occurs in the absence of Ca^2+^ and with Ba^2+^ as charge carrier (for reviews see Ben-Johny et al., 2015; Hardie and Lee, 2016). For L-type channels the differential effects on Ca^2+^- and voltage-dependent inactivation have been quantified for some mutations, showing that this pronounced slowing of inactivation is mainly due to reduced voltage-dependent inactivation (Splawski et al., 2004; Pinggera et al., 2018). Why can we be confident that this type of macroscopic gating change in HEK-293 cells can also translate to enhanced cellular Ca^2+^ signaling *in vivo*? As mentioned below, a type 1 gating change has been reported for Cav1.2 channels in LQT8 and explains the prolongation of the cardiac action potential driven by the failure of the channel to inactivate (Yazawa et al., 2011; Drum et al., 2014; Estes et al., 2019). Moreover, in Cav1.3 channels type 1 gating is associated with ASD (G407R, Pinggera et al., 2015) and a more severe neurodevelopmental syndrome (G403D, Scholl et al., 2013; Figure 3A) which cannot be explained by a Cav1.3 channel LOF (Ortner and Striessnig, 2020). In addition, the GOF phenotype is supported by somatic mutations in Cav1.3 (G403R,G403D) in aldosterone-producing adenomas (APAs), in which enhanced Ca^2+^ entry is known to drive excess Ca^2+^-dependent aldosterone production (Boulkroun et al., 2020). When expressed in HEK-293 cells, some type 1 mutations result in smaller current amplitudes but, due to slow inactivation, mutant currents exceed the amplitude of wildtype currents upon prolonged depolarization (Figure 2A, right panel, dashed line; Pinggera et al., 2018). Accordingly, expression of such a type 1 Cav1.3 channel mutant (G407R) in cultured GLT muscle cells enhanced intracellular transients (Pinggera et al., 2015).

**FIGURE 4:**
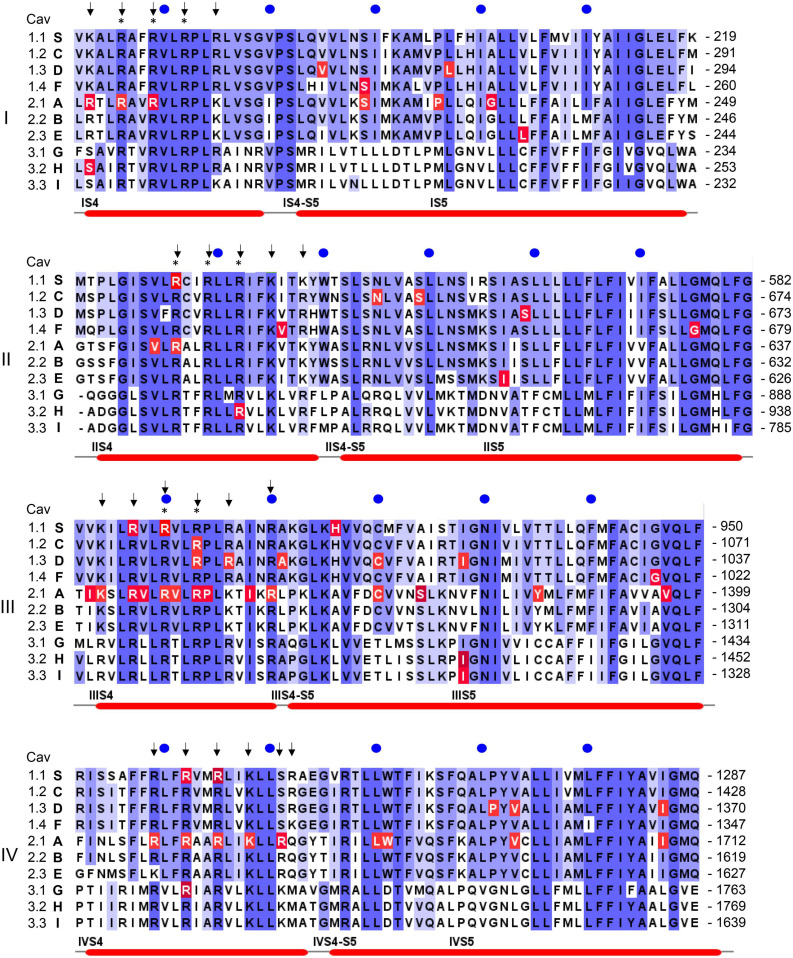
Position of pathogenic mutations within S4, the S4-S5 linkers and S5 helices of the ten different Ca^2+^ channel α1-subunits. The sequence alignment for the human α1-subunits was generated using Clustal omega and Jalview software (www.jalview.org; Waterhouse et al., 2009). Conserved residues are highlighted in blue. The accession numbers are identical to the alignment in Wu et al. (2016): *CACNA1S* (S, Cav1.1): Q13698; *CACNA1C* [C, Cav1.2; contains exon 8 (Splawski et al., 2005)]: Q13936; *CACNA1D* (D, Cav1.3): Q01668; *CACNA1F* (F, hCav1.4): O60840; *CACNA1A* (A, Cav2.1): O00555; *CACNA1B* (B, Cav2.2): Q00975; *CACNA1E* (E, hCav2.3): Q15878; *CACNA1G* (G, hCav3.1): O43497; *CACNA1H* (H, hCav3.2): O95180; *CACNA1I* (I, hCav3.3: Q9P0 × 4. All amino acid positions in the text are numbered according to the above Uniprot accession numbers and may differ in the original publications or other databases. Arrows indicate the position of positively charged residues (gating charges) in S4 helices, asterisks indicate the presence of pathogenic gating pore mutations in human Nav1.4 (*SCNA4*) or Nav1.5 (*SCNA5*) Na^+^-channels. Numbers on the right indicate the amino acid position of the last residue in each line, blue circles are 10 residues apart. Red lines on the bottom denote secondary structural elements of the rabbit Cav1.1 channel (Wu et al., 2016) with labels placed at the start of a feature. Residues affected by a mutation are highlighted in red. Note that not all highlighted mutations are discussed in the text. For information on mutations in all Ca^2+^ channels refer to the Uniprot-database and to reviews cited in the text.

Figure 2B illustrates another typical pathological pattern, termed “type 2” for further discussion. Its characteristic feature is a shift of the activation voltage range to more negative voltages. The steady-state inactivation voltage range is either unchanged (e.g., Cav1.3-V401L, Cav2.1-S218L; Tottene et al., 2005; Pinggera et al., 2017) or is shifted to more negative potentials (e.g., Cav1.3-S652L, Cav3.1-A961T, Cav3.2-M1549V; Scholl et al., 2015; Chemin et al., 2018; Hofer et al., 2020) (Figures 3, 4). As a consequence, the voltage-range at which channels can conduct a steady-state Ca^2+^ inward current (the so-called “window current”, i.e., the voltage range at which the steady-state activation and inactivation curves overlap) is also shifted to more negative voltages (see legend to Figure 3B). The time course of Ca^2+^-current inactivation may be accelerated (e.g., Cav1.3-S652L, Cav2.1-V714A; Kraus et al., 1998; Hofer et al., 2020) or slowed (Cav1.3 –V401L, Cav2.1-S218L, Cav3.1-A961T, Cav3.2-M1549V; Tottene et al., 2005; Scholl et al., 2015; Pinggera et al., 2017; Chemin et al., 2018). A slowing of current deactivation upon repolarization can also occur but is not illustrated here (Cav1.3-S652L, Cav1.3-A749G, Limpitikul et al., 2016; Hofer et al., 2020) (Figures 3, 4). In L-type channels, this type of gating change appears to be associated with reduced Ca^2+^-dependent inactivation (as shown for Cav1.3 mutations V401L, S652L, A749G; Limpitikul et al., 2016; Pinggera et al., 2017; Hofer et al., 2020). While the negative shift of activation voltage is compatible with a GOF, more negative steady-state inactivation and the faster current inactivation time course can can lead to reduced channel activity. This LOF will be mainly relevant in cells, which, on average, rest at a membrane potential positive enough to allow more channels to inactivate along the more negative steady-state inactivation voltage-dependence. In addition, the faster inactivation time course would mainly be relevant if the cell’s firing pattern involves prolonged depolarized states. Therefore, it appears that more negative activation and window current are sufficient to induce GOF disease phenotypes, like in Cav2.1 for Familial hemiplegic Migraine Type I (FHM1; e.g., V714L, T501M, Hans et al., 1999; Carreño et al., 2013), in Cav1.3 for severe neurodevelopmental disorders (e.g., S652L, A749G, Ortner and Striessnig, 2020), in Cav1.2 for Timothy Syndrome (e.g., I1166T, Boczek et al., 2015) and in other Cavs (see below).

Type 3 changes refer to slowing of the inactivation time course alone as a GOF feature without detectable changes in the voltage-dependence of gating (Figure 2C) and has also been found for some pathogenic mutations (e.g., Cav1.3-P1336R, Azizan et al., 2013).

In addition to macroscopic gating changes, protein-coding missense variants can also alter ion permeation through the channels by affecting unitary conductance or ion selectivity. Especially mutations close to the channels’ selectivity filter can alter single channel conductance, open probability and the voltage-dependence of single channel gating. The selectivity filter is formed by four conserved negatively charged residues (red dots in Figure 1B) held in place by two supporting pore helices (P1, P2 in Figure 1B, Wu et al., 2016; Catterall et al., 2020) in the S5-S6 linker of each domain. This has been studied for the Cav2.1 FHM1 variant T666M (2 residues N-terminal of the selectivity filter glutamate; Hans et al., 1999; Tottene et al., 2002). Mutations in the activation gate formed by the cytoplasmic ends of the S6 helices also affect ion selectivity and single channel conductance (e.g., Cav2.1-V714L, Cav1.4-I756T in IIS6; Figure 3; Hans et al., 1999; Tottene et al., 2002; Williams et al., 2020). A dramatic effect on ion selectivity has recently been reported for *CACNA1F* mutation I756T (Figure 3), which depends on C-terminal splicing and the associated β-subunit isoform (Williams et al., 2020).

Pathogenic variants directly hitting the negative charges forming the Ca^2+^ selectivity filter are rare but have been reported for Cav1.2 (E1135K) associated with LQT8 and Cav2.1 associated with a Spinocerebellar Ataxia Type 6 (SCA6) phenotype (not illustrated; see below). This can create a channel population permeable for monovalent cations with Na^+^-mediated inward currents and K^+^-mediated outward currents. It can explain the prolongation of the cardiac action potential in cardiomyocyte-like cells derived from human induced pluripotent stem cells expressing the E1135K LQT8 variant (Ye et al., 2019).

### Pathogenic Gating Pore Currents

**FIGURE 5:**
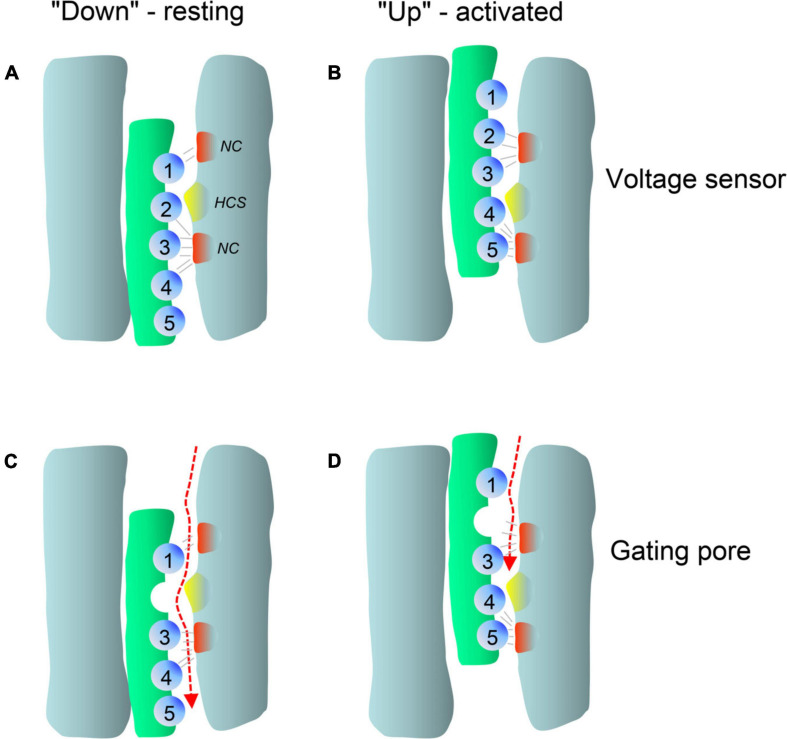
Membrane potential dependent conformations of the voltage-sensor allow pathogenic gating pore current. Simplified scheme illustrating the membrane potential dependent conformations of a single voltage-sensor and the effects of a mutation in gating charge R2. Membrane-spanning S4 helices are shown in green, positively charged residues (mostly arginines spaced at three residue intervals) as blue spheres. Clusters of extracellular and intracellular negative counter-charges (NC) forming ion pair interactions with the gating charges are shown in red. The hydrophobic constriction site is indicated in yellow (HCS). **(A)** In the resting state, the positively charged S4 helix is pulled inside by the negative resting potential (**A**, S4 “down”). **(B)** Upon depolarization, the S4 segment moves outward according to a sliding-helix model (Catterall et al., 2020; **B**, S4 “up”), and transports positive gating charges through the HCS. Inside and outside the HCS the arginine side chains are stabilized by forming ion pairs with negative charges within the VSD (gray lines). Upon S4 movement the ion pair partners are exchanged and the large arginine side chains seal the VSD and prevent formation of a water filled space through which ions can flow (Catterall et al., 2020). Replacement of a positive gating charge by a smaller or hydrophilic residue can disrupt this seal as exemplified here for a neutralizing mutation in R2 **(C)**. This position of the mutation permits an inward gating-pore current during the hyperpolarized “down”-state of the S4 helix at negative voltages but not during the depolarized “up”-state at positive voltages **(D)**. In contrast, mutations of one of the inner gating charges would permit gating pore current to flow in the activated but not the resting position of S4 (for X-ray structures of mutant voltage-gated Na^+^-channels see Jiang et al., 2018; Jiang D. et al., 2019).

In addition to altering the ion flow through the canonical central pore, missense mutations can even generate a new ion-conducting pathway theoretically within each of the four VSDs (Figure 1C; Cannon, 2017; Groome et al., 2017; Jiang et al., 2018; Männikkö et al., 2018; Jiang D. et al., 2019). Whereas the pore-lining S6 helices of all four homologous α1-subunit domains together form the single, central Ca^2+^-selective pore (orange helices in Figures 1C,D), the voltage-sensing apparatus consists of four separate but cooperatively gating VSDs (Pantazis et al., 2014; Catterall et al., 2020). Each of the VSDs exists as a four-helix bundle (S1-S4 helices; Catterall et al., 2020). The S4 helix contains at least 4 positively charged arginine or lysine residues (gating charges, Figure 5; arrows in Figure 4) spaced at three (or four) residue intervals. These charges sense changes in transmembrane voltage and depolarization drives them outward across the transmembrane electric field (Figure 5; Catterall et al., 2020). The intracellularly connected S4-S5 linkers (illustrated for domain I in Figure 1D) open the pore by transmitting the S4 movements to the so-called “activation gate”, the narrow part of the pore formed by the S6 helices on their cytoplasmic ends (Figures 1C,D; Wu et al., 2016; Catterall et al., 2020). While moving through the membrane, the S4 positive charges are stabilized by ion pairs formed with negative countercharges from neighboring helices (NC in Figure 5) in- and outside of a hydrophobic constriction site (HCS in Figure 5) whereby ion pair partners are exchanged dynamically during this “sliding helix movement” (Figure 5; Wu et al., 2016; Catterall et al., 2020). The HCS helps to shield the structure from the extracellular hydrophilic milieu and prevents transmembrane flux of water and ions through the VSD. Positive charges are stabilized around the HCS and thereby also prevent ion flux (Figure 5). However, if one of these positive charges is mutated and replaced by a smaller or hydrophilic amino acid this seal can open, allowing water molecules to enter. The resulting pore can be large enough to conduct either measurable Na^+^ and K^+^ currents or proton currents if a positive gating charge is replaced by a protonable histidine (Figure 5). When open, this artificial pore (“gating-pore” or “ω-pore”) can conduct outward or inward current depending on the driving forces imposed by transmembrane voltage. Depending on the position of a mutation within the S4 helix a “gating pore current” can develop in the resting state when the outermost gating charges are affected. As illustrated in Figure 5, these residues are close to the HCS when the S4 helix is “down” and, when mutated, allow formation of a water wire through which ions can pass. In contrast, inner gating charges are close to the HCS when the S4 helix is “up.” Their neutralization can support gating pore current when S4 is “up,” which is the case during the open and inactivated state until repolarization forces S4 helices again into their “down” state (Sokolov et al., 2008; Cannon, 2017; Groome et al., 2017; Jiang et al., 2018; Männikkö et al., 2018; Jiang D. et al., 2019). Notably, also variants outside the S4 helix can induce gating pore currents by inducing conformational rearrangements within the VSD (Fuster et al., 2017; Monteleone et al., 2017; Flucher, 2020; see section on *CACNA1S* below).

In addition to pathogenic gating pore current, S4 mutations can also impair voltage-sensor movement thus affecting channel gating and Ca^2+^ inward current through the canonical central pore (Jurkat-Rott et al., 2012). However, in the case of Periodic Paralysis mutations in Cav1.1 Ca^2+^- (*CACNA1S*) and Nav1.4 Na^+^- channels (*SCN4A*) only the gating-pore currents are disease-relevant (see section on *CACNA1S* below). The function and structure of VSDs is highly conserved among voltage-gated ion channels, including Cavs, Na^+^- (Navs) and K^+^-channels. Therefore, S4 mutations in Cav1.1 or in Nav1.4 VSDs can both cause Periodic Paralysis in humans (Cannon, 2017; Groome et al., 2017; Jiang et al., 2018). Notably, this has important implications also for the discussion of the functional consequences of variants found in other Ca^2+^-channelopathies, since VSD mutations in the same conserved position of different Cavs may cause similar gating-pore currents and perhaps even similar phenotypes. Indeed, when discussing a number of gating charge mutations for the individual channels below, we need to consider the possibility that gating pore currents may explain the pathogenic phenotype better than alterations of macroscopic Ca^2+^ currents (e.g., Cav1.3 – R990H, Cav3.1 – R1715H, Cav3.2 R890H; Figure 4). As a take home message, for gating charge missense variants the possibility of a gating pore current leading to abnormal channel function should always also be considered as a pathogenic mechanism independent of the mutation’s effect on the macroscopic Ca^2+^ current.

### Other Mechanisms

Especially for variants outside the classical regions responsible for ion permeation, gating and voltage-sensing other molecular pathogenic mechanisms have to be considered. This includes sites for protein interactions with one of the channel’s accessory subunits or with other proteins. Several examples exist: the (expected) LOF phenotype of the Episodic Ataxia *CACNA1A* variant L437F may be explained by reduced affinity for β-subunits (which are essential for channel function, Buraei and Yang, 2010), because this mutation occurs within the α1-subunit’s cytoplasmic I-II linker where β-subunits bind (Van Petegem et al., 2004). In LQT8 families Cav1.2 channel variants were found to cluster within four-residues (857-860, a PRPR-motif, not illustrated) in the cytosolic II–III loop region and enhance channel activity through a so far unknown mechanism. Recently, crystal structures and homology modeling provided compelling evidence that this region binds SH3-domain-containing proteins (e.g., STAC) and LQT8 variants are expected to weaken this interaction (Mellor et al., 2019). Another example is the slow inactivation of the Cav1.2-G406R type 1 mutation, which appears to be mediated by cyclin-dependent kinase 5 (CDK-5; Song et al., 2017).

These examples highlight another weakness of heterologous expression systems in which mutations affecting protein-protein interactions are less likely to be detected.

## Ca^2+^-Channelopathies

How do these GOF or LOF mechanisms translate into different clinical syndromes? As mentioned above, we only focus on disorders in which rare single missense variants explain high disease risk. We will not discuss variants weakly contributing to polygenic disease risk.

### Cav1.1 (*CACNA1S*)

Cav1.1 is the only relevant Cav species expressed in adult skeletal muscle. Due to the slow activation kinetics of the adult splice variant and its positive activation voltage-range (Tuluc and Flucher, 2011) Ca^2+^ entry through the channel does not directly contribute to fast excitation-contraction coupling in skeletal muscle (Zamponi et al., 2015). Instead, fast conformational changes of its VSDs open the ryanodine receptor and induce fast SR Ca^2+^ release through the cytoplasmic linker connecting domains II and III (Figure 1) and the associated essential EC-coupling protein STAC3 (for review see Flucher and Campiglio, 2019).

Heterozygous LOF mutations in the Cav1.1 α1-subunit appear clinically silent, unless variants on the second allele reduce function further. This is the case in recessively inherited *CACNA1S*-associated **Congenital Myopathy** (Schartner et al., 2017; Flucher, 2020), a disorder characterized by marked neonatal hypotonia, generalized weakness with pronounced axial involvement, variable symptoms such as respiratory and swallowing issues, delayed motor development and peculiar histological features in muscle biopsies (Schartner et al., 2017). Interestingly, *heterozygous*, dominantly inherited protein-coding missense mutations were also identified in 3 unrelated families located in functionally sensitive regions of the channel (with P742Q and P742S in the II-III linker; not illustrated) expected to weaken channel coupling to the ryanodine receptor (Schartner et al., 2017). Like with recessive mutations, Western blots of patient muscle biopsies showed that the dominant missense variants also induced a strong decrease in overall Cav1.1 protein expression below a level expected from haploinsuffiency. This suggests a dominant-negative effect on wildtype protein expression (Schartner et al., 2017). It is therefore tempting to speculate that, as described for *CACNA1A* LOF variants (see section on *CACNA1A* below; Mezghrani et al., 2008), this may result from the interaction of wildtype with mutant Cav1.1 α1-subunits and the formation of complexes susceptible to early protein degradation.

As mentioned above, *CACNA1S* variants causing **Hypokalemic** (HypoPP1, familial autosomal dominant inheritance or sporadic *de novo*) and **Normokalemic Periodic Paralysis** (NormoPP, Table 1) are the most relevant also for our understanding of the potential functional effects of gating charge mutations in other Cav isoforms. HypoPP1 mutations affect arginines of S4 voltage sensors in domains II–IV and produce an anomalous gating pore leak current (the positions of the mutations are highlighted in red in Figure 4). Like gating pore currents in Nav1.4, these currents, and not changes in the function of the central pore, cause attacks of weakness in HypoPP1 (Cannon, 2017; Flucher, 2020). Seven of the 9 known variants affect arginines. Exceptions are V876E in IIIS3, which also produces gating pore currents (Fuster et al., 2017; Monteleone et al., 2017) and H916Q in the IIIS4-S5 linker (Flucher, 2020; Figure 4). In addition to *CACNA1S* arginine mutations (Flucher, 2020), gating pore currents have so far only been investigated and confirmed experimentally for Cav1.3-R990H, which therefore is expected to induce a GOF phenotype (see section on *CACNA1D* below) despite a LOF gating change (Monteleone et al., 2017). It is therefore possible that S4 gating charge variants in other Cav isoforms also generate disease-relevant gating pore currents. Figure 4 illustrates that outside of Cav1.1 α1, disease-associated mutations were reported in a total of at least 17 S4 gating charges in 5 different Cav isoforms (Cav1.2, Cav1.3, Cav2.1, Cav3.1, Cav3.2). Many of them are located in the same homologous positions as Cav1.1 HypoPP1 mutations or pathogenic Nav1.4 (HypoPP2) or Nav1.5 (arrhythmias and dilatated cardiomyopathy) mutations (indicated by asterisks in Figure 4; Groome et al., 2017; Jiang D. et al., 2019; Kubota et al., 2020).

For missense variants causing other skeletal muscle disorders (**Malignant Hyperthermia Susceptibility**) the disease-inducing mechanism is less well understood. Malignant Hyperthermia Susceptibility mutations sensitize RyR1 Ca^2+^ release to activation by caffeine and volatile anesthetics but their location in different regions of the channel currently provides no clue about the molecular mechanism (see reference Flucher, 2020 for details).

### Cav1.2 (*CACNA1C*)

Cav1.2 GOF mutations can cause both a syndromic (Timothy syndrome, TS) and non-syndromic form of long-QT syndrome (LQT8). In contrast, LOF mutations shorten the cardiac action potential and are associated with Brugada syndrome and short-QT syndrome (LQTS; Antzelevitch and Yan, 2015; Priori et al., 2015; Antzelevitch et al., 2017). Excellent articles have reviewed this topic recently (Antzelevitch et al., 2017; Marcantoni et al., 2020). Like in other Cavs, GOF mutations are located preferentially in the functionally most sensitive regions of the gating apparatus, the S4 helices, the coupling machinery of the S4-S5 linker and the activation gate formed by the S6 helices (Figure 1D). Examples for regions affected by mutations in different Cavs are indicated in blue in Figure 1D.

**Timothy syndrome** is a rare systemic disorder originally described as the *heart-hand syndrome* (Reichenbach et al., 1992). This refers to the typical diagnostic findings of LQTS, often leading to fatal arrhythmias at young age, and unilateral or bilateral cutaneous syndactyly of fingers or toes. In surviving individuals other characteristic clinical features were described compatible with a multi-organ disease. In most cases facial anomalies (including depressed nasal bridge, low-set ears, thin vermilion of the upper lip), congenital heart defects and developmental delay (language, motor, and generalized cognitive impairment) are present. Autism, seizures, and intellectual disability as well as hypoglycemia were also reported (Splawski et al., 2004). These symptoms are in accordance with the known role of Cav1.2 for cardiac function, neuronal development and plasticity (Krey et al., 2013; Zamponi et al., 2015), craniofacial development (Ramachandran et al., 2013), and insulin secretion (Sinnegger-Brauns et al., 2004; Zamponi et al., 2015).

Classical TS is caused by GOF mutations in Cav1.2. Originally, only mutations in alternatively spliced exons 8a (G406R, **TS1**) and 8 (G406R, G402S, **TS2**) had been described (Splawski et al., 2004; Splawski et al., 2005) (Figure 3; exon 8a sequence). All of them cause GOF by a pronounced reduction of (voltage-dependent) channel inactivation (type 1, Figure 2A). This can explain a sustained depolarizing Ca^2+^ influx with broadening of the action potential and QT-prolongation. A more severe LQT phenotype (QTc > 620 ms) has been reported in TS2. This was explained by the higher abundance of exon 8 in heart and brain. Interestingly, the first two TS2 patients had no syndactyly, one of the diagnostic features of the syndrome, but were affected by facial dysmorphism and developmental delay. The phenotype in the patient with the G402S mutation was milder. It was attributed to somatic mosaicism with the mutation being much less abundant in oral mucosa than in blood DNA (Splawski et al., 2005). This was an important observation because it shows that the severity of the phenotype is also determined by mutation load. Mosaicism is also evident from severely affected TS1 and TS2 cases who inherited the mutation from a parent with germline mosaicism. The parent had either no symptoms (Splawski et al., 2004), or, upon full medical work-up, revealed diagnostic features, such as syndactyly with and without LQT8 (Etheridge et al., 2011; Dufendach et al., 2013; Fröhler et al., 2014; Baurand et al., 2017). Therefore mosaicism has to be considered in the interpretation of phenotype-genotype relationships (Lim et al., 2017; Myers et al., 2018; Cao et al., 2019) not only of *CACNA1C*-associated disorders but also of other Ca^2+^-channelopathies (e.g., *CACNA1A*, Cao et al., 2019; see below).

In addition to the classical TS mutations, four mutations outside exon 8 and 8a were reported. A GOF phenotype has either been proven in functional experiments or can be assumed based on their locations in S6 or the S4-S5 linkers. The affected individuals presented as atypical TS with LQT8 but some other clinical features of TS missing, such as syndactyly (S643F, type 1; Figure 4; Ozawa et al., 2018), or facial dysmorphisms (E407A, adjacent to G406R, Figure 3; Colson et al., 2019). In other cases, an even broader phenotypic spectrum was reported, including osteopenia, cerebral and cerebellar atrophy, intractable irritability (I1186T, type 2; Figure 3; Boczek et al., 2015) or intractable seizures and stroke (A1521G, type 2, Figure 3; Gillis et al., 2012).

Two additional variants deserve attention because their clinical phenotypes are even more distinct, R1024G and E1135K: R1024G (Figure 4) was reported in a 5-year-old girl with profound developmental and speech delay, TS-like craniofacial dysmorphism and syndactyly but *without* LQTS upon repeated ECG measurements (Kosaki et al., 2018). Pulmonary hypertension was also diagnosed. Although no functional studies have been performed, the position of this variant is intriguing. It neutralizes one of the positive gating charges in voltage sensor IIIS4. It corresponds to the R990G and R990H mutations in Cav1.3 (Figure 4; somatic mutations in APAs, see section on *CACNA1D* below). In Cav1.3 R990H destabilizes the VSD and can conduct a depolarizing gating pore current at negative membrane potentials (Monteleone et al., 2017). VSD mutations R1135H and R1135C in Nav1.4, which cause HypoPP2 (see above, position indicated by asterisk in Figure 4C) also occur in the equivalent positions. One possible scenario therefore is that R1024G also induces a gating pore current in Cav1.2. Assuming that it destabilizes the VSD in a similar manner as R990H in Cav1.3 it could contribute depolarizing current at more negative potentials. In arterial smooth muscle cells increased arterial muscular tone could explain pulmonary hypertension, whereas in neurons enhanced subthreshold neuronal depolarization could drive abnormal neuronal firing underlying the severe neurodevelopmental phenotype. However, upon activation of the Cav1.2 voltage-sensors (S4 “up” in open and inactivated channels) during the plateau of the cardiac AP, this gating pore current is expected to turn off and may thus not affect AP duration and QT-interval.

E1135K (not illustrated) was found in a 14-year-old male proband with idiopathic QT prolongation, autism spectrum disorder and unexplained hyperglycemia (Ye et al., 2019). This mutation reverses one of the four essential negative charges of the channel’s selectivity filter. Functional expression in tsA-201 cells confirmed the expected conversion of a Ca^2+^-selective channel into a *non-selective* cation channel with marked increase in both peak and persistent inward Na^+^- and outward K^+^-currents (Ye et al., 2019). Its expression in human iPSC-derived cardiomyocytes prolonged the cardiac AP. By favoring membrane depolarization, it can therefore be considered a GOF variant in the heart and likely also in neurons, which could affect neuronal excitability and underlie the autism phenotype. The mutation may also account for the “unexplained” hyperglycemia reported in this patient due to reduced insulin secretion. An increased membrane depolarization of the pancreatic β-cell could result in an increased basal intracellular Ca^2+^ level and reduced glucose-induced insulin secretion as observed upon genetic deletion of the K_ATP_ K^+^-channel subunits (Miki et al., 1998). Alternatively, while mediating a non-selective pore current, the mutated channels are not expected to conduct Ca^2+^. This could impair Ca^2+^-dependent processes depending on Cav1.2, including insulin secretion (Zamponi et al., 2015; Mastrolia et al., 2017).

In addition to TS, GOF Cav1.2 mutations can also cause **a non-syndromic form of LQT8**. LQT8 can be regarded as part of a *CACNA1C* disease spectrum because with some variants the clinical phenotype can be either LQT8 alone (G402S, Mellor et al., 2019), LQT with syndactyly but without other TS-typical features (G402S, I1186T; Fröhler et al., 2014; Wemhöner et al., 2015) or Timothy syndrome. Somatic mosaicism and/or genetic background can explain these variable phenotypes. Many of the LQT8-associated variants were confirmed to induce GOF (Hennessey et al., 2014; Fukuyama et al., 2014b; Boczek et al., 2015; Wemhöner et al., 2015; Landstrom et al., 2016; Chavali et al., 2019; Estes et al., 2019) with slowing of the inactivation time course and increased late currents. Introduction of LQT8 mutations into human iPSC-derived cardiomyocytes confirmed the broadening of the action potential in the heterozygous state (Estes et al., 2019; Chavali et al., 2019). It is possible that mutations shifting the voltage-dependence of activation and inactivation to more negative voltages favor more severe extracardiac manifestations (in particular the nervous system; Marcantoni et al., 2020). However, some variants may predominantly affect the function of cardiac channels. This has recently been shown for a cluster of variants (affecting residues P857, R858, R860; not illustrated) located within the long cytoplasmic II-III – linker without obvious role in channel gating and conductance. However, this proline-rich region serves as a binding site for SH3-domain containing proteins, such as STAC proteins, known to affect the expression and function of L-type channels (Mellor et al., 2019; Flucher and Campiglio, 2019). For a complete list of LQT8 mutations see recent reviews (Giudicessi and Ackerman, 2016; Zhang et al., 2018; Marcantoni et al., 2020).

LOF *CACNA1C* variants are associated with so-called J-wave syndromes (named after the typical electrocardiographic “J-wave”), consisting of the **Brugada syndrome** and **short-QT** syndromes (Priori et al., 2015; Antzelevitch and Yan, 2015; Antzelevitch et al., 2017). Variants leading to altered Cav1.2 activity in the heart were also reported in β- (*CACNB2* gene) and α2δ1 (*CACNA2D1*) subunits (Antzelevitch et al., 2007; Burashnikov et al., 2010). LOF *CACNA1C* variants are protein-coding variants predominantly located in the cytoplasmic regions of the channel and it is less clear how they induce the observed reduction in Ca^2+^ current when heterologously expressed. Synonymous variants in splice sites inducing nonsense-mediated RNA-decay have also been described (Fukuyama et al., 2014a). Interestingly, unlike with pathogenic *CACNA1F* and *CACNA1A* LOF mutations (see below), *no protein-truncating variants* have been reported so far. This could indicate that these phenotypes are not simply due to haploinsufficiency.

### Cav1.3 (*CACNA1D*)

Current evidence from knockout mouse models and human genetics indicate that heterozygous Cav1.3 LOF is unlikely to cause a disease phenotype. About 20 heterozygous LOF genotypes^2^ are listed for mostly healthy control individuals in the gnomAD database. Only homozygous Cav1.3-deficiency causes sinoatrial node dysfunction and deafness in mice (Platzer et al., 2000; Zamponi et al., 2015). The same phenotype (**Sinoatrial Node Dysfunction and Deafness**, Table 1) is observed in humans with a homozygous LOF mutations in exon 8 (also referred to as exon 8b; Baig et al., 2011), which is the predominant splice variant in brain, sinoatrial node and the cochlea (Baig et al., 2011; Zamponi et al., 2015; Liaqat et al., 2019).

In contrast, heterozygous *de novo* GOF missense mutations cause human disease by affecting multiple Cav1.3-dependent physiological functions. Since we have recently reviewed the disease spectrum associated with *CACNA1D* GOF variants (Ortner and Striessnig, 2020), this is only briefly summarized here.

In most affected individuals germline missense DNMs lead to a **severe neurodevelopmental disorder** with ID, developmental delay, seizures, auto-aggressive behaviors, delayed or no speech development, muscle hypotonia and autism spectrum disorder (V401L, S652L, A749T; Figure 3). In even more severe cases congenital hyperinsulinemic hypoglycemia and/or congenital aldosteronism (Ortner and Striessnig, 2020) can be present at birth (V259A, G403D, I750M, L271H; Figures 3, 4). A syndrome, **Primary Aldosteronism with Seizures and Neurological Abnormalities (PASNA)** is part of this disease spectrum. Less severe cases were diagnosed with ASD with (A749G) and without (G407R) mild intellectual impairment (Figure 3). So far, for these Cav1.3 – linked disorders type 1 and 2 GOF gating changes were reported for nine mutations in 12 individuals (Ortner and Striessnig, 2020) but unpublished cases with pathogenic mutations exist and are currently being characterized. For a detailed list of mutations and associated phenotypes see Ortner and Striessnig (2020). Like most other GOF mutations, they are located in the activation gate (cytoplasmic region of S6 helices) as well as in the S5-S6 linkers, see above and Figures 1D, 3, 4). Interestingly, the positions of G403R and G403D (exon 8) and G407R (exon 8a) are identical to the Timothy syndrome mutations G402 and G406 (see above) in *CACNA1C* and also cause very similar gating defects (type 1; Scholl et al., 2013; Pinggera et al., 2018). When present as *somatic* mutations in **APAs**, these variants induce excessive Ca^2+^-dependent aldosterone production resulting in drug-resistant hypertension (Azizan et al., 2013; Scholl et al., 2013). Some of these mutations affect the same amino acid or are even identical to the ones identified germline. Importantly, some of the germline mutations exhibit *increased* sensitivity to DHP LTCC blockers (Hofer et al., 2020). Future clinical research should therefore test the potential of these drugs to improve symptoms in severely affected individuals. Preliminary evidence suggests that muscle hypotonia may respond to treatment with DHPs (De Mingo Alemany et al., 2020).

### Cav1.4 (*CACNA1F*)

Cav1.4 L-type calcium channels are essential for tonic glutamate release from retinal photoreceptors (as is Cav1.3 in cochlear inner hair cells; Zamponi et al., 2015; Pangrsic et al., 2018). Their α1-subunit gene (*CACNA1F*) was originally discovered as the target of LOF mutations causing **X-linked** C**ongenital Stationary Night Blindness Type 2 (CSNB2)**, a non-progressive vision disorder with characteristic clinical findings (Zamponi et al., 2015). Since then a large number of *CACNA1F* LOF mutations, most of them protein-truncating variants, have been reported to be associated with CSNB2 as well as other eye disorders, including **Åland Island Eye Disease, Cone-Rod Dystrophy, X-linked retinal disorder**, and **Night Blindness-Associated Transient Tonic Downgaze**, which share a variety of clinical symptoms (Hope et al., 2005; Stockner and Koschak, 2013) (Table 1). As expected for an X-linked disorder females are rarely affected. The absence of the channel protein or the absence of functioning channels leads to abnormal cellular organization of synapses within the retina and defective neurotransmission between photoreceptors and second-order neurons (Zamponi et al., 2015).

Interestingly, a number of missense variants have also been identified. While some of them decrease channel function or expression (Hoda et al., 2006; Stockner and Koschak, 2013), others show the typical gating changes compatible with a gain of channel function. For example, G369D in domain I and F753C and I756T in the activation gate of domain II (Figure 3) cause a dramatic type 2 gating change and slow inactivation as expected from their locations (Hemara-Wahanui et al., 2005; Hoda et al., 2005; Peloquin et al., 2007). One plausible explanation of how these gating changes can actually decrease Cav1.4 channel activity is that the pronounced negative shift of voltage-dependent activation gating moves the channel out of its optimal operating range within the voltage window during light exposure (about − 55 mV) and darkness (up to −36 mV). During illumination a larger fraction of mutated Cav1.4 channels may already be activated thus limiting the maximal gain during depolarization in the dark (Stockner and Koschak, 2013). This is another example demonstrating that a given “GOF” gating change can either increase or decrease channel signaling depending on the context of a cell’s electrical activity pattern (see above).

### Cav2.1 (*CACNA1A*)

Cav2.1 channels are widely expressed in the central (including thalamocortical, cortical, cerebellar neurons) and peripheral nervous system. Together with Cav2.2 channels they are tightly coupled to fast neurotransmitter release at presynaptic terminals, including motoneurons (Zamponi et al., 2015). Homozygous knockout mice develop rapidly progressive neurological symptoms with ataxia and dystonia and die a few weeks after birth (Jun et al., 1999; Fletcher et al., 2001). Therefore, homozygous LOF of Cav2.1 is also expected to be incompatible with life in humans. Accordingly, no homozygous *CACNA1A* pLOF variants are reported in the gnomAD database.

*CACNA1A* mutations cause several dominantly inherited or sporadic neurologic disorders with a wide spectrum of neurological symptoms (Spacey, 2015): hemiplegic migraine with aura (including severe forms with cerebral edema and coma), cerebellar signs (including episodic, permanent, early or late onset progressive ataxia, cerebellar atrophy, nystagmus, vertigo, dysarthria), seizures, and developmental delay. Although symptoms often overlap, the three main neurological *CACNA1A* disorders reveal some genotype-phenotype correlation. **Episodic ataxia type 2 (EA-2)** typically starts in childhood or early adolescence and is characterized by paroxysmal attacks of ataxia and other cerebellar signs and can be associated with other symptoms including hemiplegia and headache (Spacey, 2015). Attacks may last from minutes to days. The majority of EA-2-associated mutations are pLOF mutations or missense mutations, which typically reduce current amplitudes and/or lead to LOF gating changes. When expressed as trunctated proteins *in vitro* some of them also exert a dominant negative effect on wildtype channels (see above). Excellent reviews summarizing the position of pLOF and missense LOF variants associated with EA-2 have been published recently (Eising and van den Maagdenberg, 2017; Tyagi et al., 2020).

In contrast, GOF mutations cause **Familial (FHM-1) or Sporadic (SHM-1) Hemiplegic Migraine Type 1**, a form of migraine with aura, which, in addition to other aura symptoms, is characterized by the presence of motor weakness, i.e. hemiparesis (Jen, 2015).

FHM-1-associated mutations mostly exhibit GOF gating changes with enhanced channel activity at more negative voltages (Tottene et al., 2002; Pietrobon and Striessnig, 2003). They are mainly located at the positions within voltage-sensors (e.g., R192Q, R195K, V581L/M, R583Q, K1343Q, R1352Q, R1667W, K1670R), the S4-S5 linkers (e.g., S2181L, C1369Y, L1682P, W1683R) and the cytoplasmic S6 activation gates (e.g., F363S, V714A, D715E, F1506S, I1810L; Figures 3, 4) where other GOF channelopathy mutations are typically located (Eising and van den Maagdenberg, 2017; Tyagi et al., 2020). Moreover, some variants associated with FHM-1 also neutralize S4 gating charges, including positions in which also Cav1.1 and Nav1.4 gating-pores were reported (e.g., R195K, R583Q, K1343Q, R1352Q; asterisks in Figure 4; note that R195K in Cav2.1 and R219K in Nav1.4 are both charge-retaining substitutions; Ducros et al., 2001; Kubota et al., 2020) and may therefore affect neuronal excitability also through gating pore currents (Jurkat-Rott et al., 2012). This hypothesis should be tested experimentally and by molecular modeling like for other Cavs (Monteleone et al., 2017; Flucher, 2020).

As a monogenic form of migraine, FHM-1 offered important insight into the pathophysiology of migraine with aura (Pietrobon and Striessnig, 2003; Goadsby et al., 2017; Brennan and Pietrobon, 2018). Human *CACNA1A* GOF mutations (R192Q, S218L; Figure 4), when introduced in mice, promote cortical spreading depression, a self-propagating wave of neuronal and glial depolarization that spreads slowly across the cerebral cortex and underlies the aura symptoms in humans (Pietrobon and Striessnig, 2003; Brennan and Pietrobon, 2018). Moreover, enhanced excitatory, but not inhibitory synaptic transmission and alterations of cortical excitatory-inhibitory balance can explain the altered sensory and pain processing in FHM1 (Tottene et al., 2019) and likely also in more common forms of migraine.

Unlike EA-2, **Spinocerebellar Ataxia Type 6 (SCA-6)** is characterized by adult onset, slowly progressive rather than episodic cerebellar ataxia, dysarthria, and nystagmus with a mean age of onset between 43 and 52 years (Du and Gomez, 2018; Casey and Gomez, 2019). Genetic diagnosis of SCA-6 requires documentation of an expanded polyglutamine repeat within the C-terminus of the Cav2.1 α1-subunit. There is strong experimental evidence that, unlike the other Ca^2+^-channelopathies described here, SCA6 is not caused by a dysfunction of the Cav2.1 channel itself, but instead by a separate protein, the transcription factor α1ACT. This protein is also encoded by the *CACNA1A* gene due to the presence of an internal ribosomal entry site (IRES) within its mRNA (for a detailed review see Du and Gomez, 2018). α1ACT is important for normal perinatal cerebellar developmental and motor function (Du and Gomez, 2018). Wildtype α1ACT, but not α1ACT-containing SCA6 poly-glutamine expansions (α1ACTSCA6), can rescue neurological deficits when transgenically expressed in Cav2.1 knockout mice (Du and Gomez, 2018). α1ACTSCA6 is also toxic to cultured cells and when expressed transgenically in Purkinje cells (Du and Gomez, 2018).

*Substantial phenotypic overlap* is observed for these three *CACNA1A* disorders, complicating the interpretation of this genotype-phenotype relationship. For example, a large fraction of FHM-1 families with GOF variants report cerebellar signs ranging from nystagmus to progressive, usually late-onset mild ataxia (Ducros et al., 2001; Jen, 1993). EA-2 individuals (i.e., with LOF variants) may also eventually develop interictal symptoms and cerebellar signs such as truncal ataxia and nystagmus. Such late-onset progressive ataxia makes the clinical phenotype similar to SCA-6 (Di Cristofori et al., 2012). In some families progressive ataxia due to missense variants and without expanded polyglutamine repeat was the major clinical feature, with some family members also diagnosed with EA-2 (Yue et al., 1997; Jen et al., 2004; Romaniello et al., 2010).

**Congential ataxia**, with presentation before the age of 2 years, is mostly associated with GOF variants, many of them also found in FHM-1 patients. Examples are S218L, D715E, I1810L, V1396M (Figures 3, 4) and T666M (close to selectivity filter in domain II, not illustrated). For an excellent detailed recent review on congential ataxia see reference Izquierdo-Serra et al. (2020).

Interestingly, a slowly progressing non-episodic spinocerebellar ataxia without migraine symptoms differing from SCA-6 and EA-2 has been associated with the mutation E668K. This mutation reverses the negative charge of the selectivity filter glutamate in domain II, similar to *CACNA1C* mutation E1135K (domain III, not illustrated) mentioned above. Non-selective ion currents and loss of Ca^2+^-conductance through this mutant may therefore contribute to the observed atypical phenotype.

*CACNA1A* mutations have also been associated with severe neurodevelopmental syndromes including early-onset epilepsy (**Early Infantile Epileptic Encephalopathy 42)** and other neurocognitive (intellectual impairment, learning disabilities) and neurological symptoms, such as ataxia, nystagmus, hypotonia (Damaj et al., 2015; Epi4K Consortium, 2016; Epperson et al., 2018; Jiang X. et al., 2019). It appears that both *CACNA1A* haploinsufficiency (like in EA-2; Damaj et al., 2015; Jiang X. et al., 2019) and FHM1-like GOF gating changes (A711T, A713T, Ser218L, Figure 3; Epi4K Consortium, 2016; Epperson et al., 2018; Jiang X. et al., 2019) can cause this phenotype. Recent studies indicate that neuropsychiatric and developmental symptoms are common in patients with episodic *CACNA1A* disorders and should be considered in the differential diagnosis of otherwise unexplained developmental delay (Indelicato et al., 2019; Humbertclaude et al., 2020).

Taken together, *in vitro* gating changes indicating increased activity of Cav2.1 at more negative voltages is mostly associated with FHM1/SHM1 and congenital ataxias, whereas channel LOF causes EA-2. However, both GOF and LOF mutations are associated with progressive ataxia and epilepsy. At present, there is no clear explanation for this observation. One hint could come from the finding that Purkinje cell-specific *Cacna1a* knockout is sufficient to cause cerebellar ataxia in mice (Mark et al., 2011; Todorov et al., 2012). This strongly suggests that reduced Cav2.1 activity is responsible for this phenotype. It is therefore possible that, as outlined above, certain gating changes can cause both, GOF (e.g., by shifting activation voltage to more negative potentials) and LOF (e.g., by shifting inactivation voltage also to more negative potentials and/or by reducing maximal channel open probability at more positive voltages) (Tottene et al., 2002). This could lead to a scenario where GOF changes predominate at e.g., cortical synaptic terminals and LOF changes at Purkinje cell bodies. At presynaptic active zones the higher open probability at threshold voltages due to type 2 gating changes could facilitate nanodomain Ca^2+^ influx and glutamate release and underlie the enhanced neuronal excitability predisposing to migraine attacks (Tottene et al., 2002). At the same time, global cellular Cav2.1 current density during action potentials at cell bodies and dendrites of cerebellar neurons may be reduced (LOF), the latter predisposing to ataxia and cerebellar neurodegeneration.

Another open question is how missense mutations inducing only a partial LOF cause EA-2. In a knock-in mouse model the EA-2-associated missense mutation F1403C (F1406C in Rose et al., 2014) reduced Cav2.1 current density in Purkinje cells of homozygous knock-in mice by about 70%, as predicted from expression in HEK-293 cells. However, this mutation failed to induce a motor phenotype as observed in other recessive *Cacna1a* mouse mutant lines with mutations resulting in similar biophysical alterations (Rose et al., 2014).

### Cav2.2 (*CACNA1B*)

Like Cav2.1-mediated P/Q-type currents, Cav2.2-mediated N-type Ca^2+^ currents also play an essential role for presynaptic neurotransmitter release. However, in contrast to Cav2.1, homozygous knockout mice show only minor abnormalities. This includes reduced pain hypersensitivity in models of inflammatory and neuropathic pain, hyperactivity, increased aggression and altered blood pressure control (Kim et al., 2009; Zamponi et al., 2015). This suggests that homozygous LOF may also not cause severe disease in humans. However, this is in contrast to recent reports, describing bi-allelic LOF variants in an autosomal recessive, complex and progressive neurologic disorder with severe neurodevelopmental delay and developmental regression, drug-resistant epileptic encephalopathy, postnatal microcephaly, hypotonia, and a non-epileptic hyperkinetic movement disorder (**Neurodevelopmental disorder with seizures and non-epileptic hyperkinetic movements**, **NEDNEH**; Gorman et al., 2019). This was confirmed in three unrelated families with different protein-truncating mutations. A possible association between a form of autosomal dominant myoclonic dystonia and the *CACNA1B* variant R1389H has also been postulated (Groen et al., 2014). However, its pathogenic potential is questionable, because it is a common variant (1:500) observed at comparable frequencies in affected and in neurologically healthy individuals (Mencacci et al., 2015). Similarly, the significance of two variants associated with adult-onset isolated focal dystonia is unclear (Cocos et al., 2020).

Taken together, current evidence suggests that heterozygous Cav2.2 LOF does not confer high risk for disease but that homozygous LOF due to protein truncating mutations can cause a severe neurodevelopmental disorder in humans.

Interestingly, unlike for other Cavs, so far no GOF variants have been reported for Cav2.2. However, rare genetic variants located within the gating-sensitive regions of Cav2.2 (Figure 1D) are reported in apparently healthy control individuals in the gnomAD database at high allele numbers. Some of these variants are even located in the identical homologous positions causing typical and disease-associated GOF gating changes in other Cavs. Variant V351E (2 residues upstream of the Timothy syndrome mutations in *CACNA1C*) is located in exactly the same position as mutation V401L (Figure 3), which causes a pronounced type 2 gating change in Cav1.3 and is associated with a severe neurodevelopmental phenotype (Ortner and Striessnig, 2020). Variants S215A and S215L affect the same serine residue in the IS4-S5 linker causing GOF changes in Cav2.1 (S218L) and Cav1.4 (S229P; highlighted in blue in Figure 1D) (Tottene et al., 2005; Hoda et al., 2005). Also, variants V1408I and N1409S in IIIS6 are surrounded by GOF variants in Cav1.2, Cav1.3, Cav2.1, Cav3.1 and Cav3.2 (Figure 3). Possible explanations are that these variants are either functionally silent or induce a LOF phenotype, which, as mentioned above, is not expected to cause disease in the heterozygous state. Alternatively, these variants may induce robust GOF changes that are not compatible with life and cause intrauterine death. Functional analysis of these variants in heterologous expression systems could answer this interesting question.

### Cav2.3 (*CACNA1E*)

Helbig and colleagues recently described *de novo CACNA1E* missense variants causing Cav2.3 GOF in 30 individuals with **Developmental and Epileptic Encephalopathies** (Helbig et al., 2018). Most affected individuals presented with pharmacoresistant seizures beginning in the first year of life, profound developmental impairment, hyperkinetic movement disorders, severe axial hypotonia and some had spastic quadriplegia. Most were non-verbal and non-ambulatory. Developmental regression occurred in 9/30 individuals often in association with seizure onset. Two recurrent variants G352R and A702T accounted for half of the cohort and with a severe phenotype. Out of the 14 variants four were functionally tested and showed type 2 gating behavior with slight slowing of inactivation. They are located within the typical regions involved in channel gating (Figures 1D, 3).

The same study also reported 3 individuals with variants introducing premature termination codons predicted to result in haploinsufficiency. These individuals had a milder phenotype, achieved independent ambulation at 15–18 months and acquired single words. Two of them had epilepsy (Helbig et al., 2018). One of them inherited the mutation from the apparently healthy father. Due to the small number of cases and the fact that a few heterozygous pLOF variants are also reported in gnomAD (“non-neuro” subset), the disease-causing potential of these variants is at present less clear.

### T-Type Ca^2+^ Channels

Based on the expression pattern of T-type channels and their special functional properties (low-voltage activated and inactivated, window current at negative voltages, recruitment upon hyperpolarization enabling low-threshold spike/rebound bursting observed in seen in many types of neurons) together with the known anti-epileptic properties of T-type channel blockers makes these channels major suspects for genetic causes of epilepsy. Cav3.1 and Cav3.2 knockout protects mice from epilepsies (Weiss and Zamponi, 2019; Lory et al., 2020). However, so far the association of most genetic variants in *CACNA1G* and *CACNA1H* and their functional mechanism of action in terms of GOF or LOF for idiopathic genetic epilepsies is less well understood (Weiss and Zamponi, 2019; Lory et al., 2020) and mutations in Cav3.1 or Cav3.2 causing high risk for epilepsy have not been described. However, changes in their activity (as shown for Cav3.1 in mice, Calhoun et al., 2016) may serve as genetic modifiers of other genetic risk factors for idiopathic genetic epilepsies. These are generally considered to have complex inheritance patterns with combinations of common with rare genetic risk variants required to cause disease. Some *CACNA1G* variants may therefore also interact with other genes, rather than being pathogenic *per se* (Weiss and Zamponi, 2019).

Excellent reviews summarizing all Cav3 channelopathies (except for *CACNA1I*, see below) have recently been published (Weiss and Zamponi, 2019; Lory et al., 2020).

#### Cav3.1 (*CACNA1G*)

*De novo* mutations A961T and M1531T were recently found in **Childhood-Onset Cerebellar Atrophy (ChCA; Spinocerebellar Ataxia Type 42 (SCA42) early-onset with neurodevelopmental deficits)** patients in unrelated pedigrees (Chemin et al., 2018; Barresi et al., 2020). ChCA is an early-onset neurodevelopmental disorder, with cerebellar atrophy, severe motor and cognitive impairments, and variable features including epilepsy and facial dysmorphisms. The mutations are located in “classical sites” of the S6 activation gates in domains II and III (Figure 2) and cause typical type 2 gating changes (Chemin et al., 2018).

In contrast, **autosomal-dominant SCA42** is characterized by a slowly progressive ataxia with an onset mainly in young adulthood. Another *CACNA1G* mutation, R1715H, has been identified in SCA42 families by different groups (Coutelier et al., 2015; Morino et al., 2015; Hashiguchi et al., 2019). When expressed in HEK-293 cells (Coutelier et al., 2015; Morino et al., 2015) it caused a positive shift of the activation and steady-state inactivation voltage with a shift of the window current to more positive voltages. A mouse animal model of SCA42 harboring this mutation confirmed these gating changes in Purkinje cells and recapitulated the phenotype (adult onset, gait instability) and pathology (Purkinje cells degeneration) of the disease (Hashiguchi et al., 2019). In contrast to R1715H knockin in mice, heterozygous and homozygous knockout of Cav3.1 in mice does not cause progressive ataxia and cerebellar atrophy indicating that the observed gating change is not a simple LOF. Again, an alternative explanation could be that this mutation affects a gating charge in the VSD of domain IV (Figure 4). While this can explain the reduced voltage-sensing of the mutant channels, it could also generate an additional gating pore current resulting in a depolarizing drive. This is also supported by the finding that R1664Q located in the homologous position in *CACNA1A* also causes an early-onset and progressive form of ataxia not characterized by the typical episodic features of EA-2 (Tonelli et al., 2006; Luo et al., 2017). Moreover, this residue corresponds to R1239 in *CACNA1S* in which R1239H- and R1239G- induced gating pore currents cause HypoPP1 (see above, 91). If the *CACNA1G* R1715H variant induces gating pore-currents it should be classified as a “GOF” variant.

Another variant, M1574K, was found in three SCA42 patients from a Chinese family but it has not been functionally characterized yet. It is located within the III-IV-linker (not illustrated) of the channel, outside the classical regions causing gating defects (Li et al., 2018). It is located within a short alternatively spliced region (exon 25 splice variant, Latour et al., 2004) and may therefore not affect all Cav3.1 channel species in the cerebellum. However, alternative splicing in this region has been found to affect Cav3.1 recovery from inactivation (Latour et al., 2004). In the structurally highly related Cav3.2 channels alternative splicing in this position (exon 25) is required for permitting the functional effects of the adjacent R1584P mutation found in the GAERS (Genetic Absence Epilepsy Rats from Strasbourg) rat model of idiopathic generalized epilepsies (Powell et al., 2009). This GOF mutation in combination with exon 25 promotes recovery from channel inactivation and enhances channel expression by disrupting the interaction of the III-IV-linker with calnexin (Proft et al., 2017). Since calnexin also reduces the expression of Cav3.1 channels (Proft et al., 2017), it is possible that the introduction of a positive charge in the M1574K variant also affects channel function and/or expression. However, this hypothesis needs to be tested in functional and biochemical studies.

#### Cav3.2 (*CACNA1H*)

Like Cav1.3, Cav3.2 is important for mediating depolarization-induced aldosterone production. As a consequence, inherited (**Familial Hyperaldosteronism Type 4**) and *de novo* germline GOF missense mutations S196L, M1549I, M1549V (Figures 3, 4), P2083L (not illustrated) (Scholl et al., 2015; Daniil et al., 2016) and R890H (Figure 4, Wulczyn et al., 2019) cause hyperaldosteronism. Neurodevelopmental symptoms (such as mild ID, social skills alterations and learning) were also reported in carriers of the M1549 variants (Daniil et al., 2016). Two somatic variants were found in **APAs** (I1430T, V1951E; Daniil et al., 2016; Nanba et al., 2020). From the five mutations reported so far, three show typical type 2 (M1549I/V) or type-3 (S196L) gating changes. I1430T corresponds to the position of the *CACNA1D* APA mutations I1015S and I1015V (IIIS5) (Ortner and Striessnig, 2020).

Unlike other mutations, R890H caused a pronounced LOF phenotype when expressed in HEK-293 cells (Wulczyn et al., 2019), a puzzling finding considering the Ca^2+^-dependence of aldosterone secretion and the other disease-causing GOF mutations in *CACNA1D* and *CACNA1H*. However, a GOF in this mutation may again be due to the induction of gating-pore currents. This is strongly supported by the observation that pathogenic gating charge mutations in the homologous positions of Nav1.4 (e.g., R675Q; Sokolov et al., 2007) and of Nav1.5 channels (R814W, Moreau et al., 2015; asterisks in Figure 4) also conduct gating pore currents.

*CACNA1H* variants V1951E and P2093L are located in the cytoplasmic C-terminus and it is less clear how they affect channel function.

#### Cav3.3 (*CACNA1I*)

Although *CACNA1I* variants seem to contribute to the polygenic risk of neuropsychiatric disorders (including autism and schizophrenia, Lory et al., 2020; Ghoshal et al., 2020), rare missense mutations causing a monogenic disorder have only been published very recently (Ghaleb et al., 2021). The *de novo* missense variant (I860N, IIS6, Figure 3) was found in a girl with a **severe neurodevelopmental disorder** with epilepsy, muscle hypotonia, hyperexcitability, severe global developmental delay, no cognitive development and progressive brain atrophy. It induces typical type 2 GOF gating properties with significantly left-shifted voltage-dependence of activation and inactivation, increased window currents, slowed activation, inactivation, and deactivation of Cav3.3 currents. Another mutation in the same position, I860M, induced less severe biophysical changes and was present in a mother and her two children with intellectual disability but no epilepsy. Two other missense mutations, I1306T (*de novo*, IIIS5, Figure 4) in a 21-month-old female and M1425I (likely *de novo*, IIIS6, Figure 2) in an 8-year old male patient were also associated with seizures, severe developmental delay, hypotonia, severe speech impairment, and cortical visual impairment. Again, all these variants affect residues in homologous positions for which cause GOF channelopathies also in other Cavs (Figures 3, 4).

Inspection of the activation gate formed by IIS6 nicely demonstrates the accumulation of pathogenic GOF mutations within this structurally and functionally highly conserved region. Together with similar aggregations of mutations in other conserved regions of the gating apparatus (Figure 1D), functionally validated pathogenic mutations in one Cav should - in combination with other ACMG critera - help to predict the level of pathogenicity of newly diagnosed variants in another Cav.

## Implications for Therapy

The appraisal of functional changes introduced by missense mutations in terms of gain- or loss-of Ca^2+^-channel function may guide the development of potential new therapies for Ca^2+^-channelopathies.

In particular, in case of pathogenic GOF mutations, specific inhibitors of the channel isoform affected by the mutation are obvious therapeutic candidates. An intriguing example was the discovery of heterozygous activating mutations in the genes encoding ATP-sensitive K^+^-channel subunits (KCNJ11, ABCC8; De Franco et al., 2020). By keeping pancreatic β-cells hyperpolarized they prevent insulin release and cause permanent neonatal diabetes and in about 20% of patients also neurodevelopmental delay (Gloyn et al., 2004; De Franco et al., 2020). In most patients switching from insulin injections to high oral doses of sulfonylureas, known well-tolerated blockers of these channels, increases quality of life, glycemic control and ameliorates some of the neurological disabilities (Ashcroft et al., 2017).

Unfortunately, not all channelopathies respond to such gene-specific pharmacotherapy. Long QT-syndromes, including LQT3 (Nav1.5 sodium channels, Arbelo et al., 2016) and LQT8 (see above), are such examples. Although LQT8 is caused by GOF mutations in Cav1.2 LTCCs, case reports of patients treated with the LTCC blockers verapamil or diltiazem for prevention of ventricular fibrillation episodes (Jacobs et al., 2006; Shah et al., 2012; Liu et al., 2016) showed mixed effects. Verapamil may not be the best choice for these patients because it is also a potent blocker of HERG K^+^-channels, favoring QT-prolongation (this explains why verapamil usually does not affect the QT-interval because it also blocks L-type channels; Zhang et al., 1999). DHP L-type channel blockers would be another option but due to their state-dependent action (with preferential inhibition of arterial smooth muscle channels and blood pressure lowering) they are only weak blockers of cardiac L-type channels (Zamponi et al., 2015). Moreover, pathogenic mutations may decrease the apparent sensitivity for DHP, as shown for nisoldipine-inhibition of the TS1 mutation G406R (3-fold increase in IC_50_; Splawski et al., 2004). Therefore, as suggested also for GOF Na^+^-channel mutations causing LQT3-syndrome (Arbelo et al., 2016), mutation-specific management may be necessary. This requires extensive characterization of the biophysical and biochemical properties of mutations (ideally in human iPSC-derived cells) to identify patient populations most likely to respond to a given channel blocker in prospective clinical trials. In contrast to the type-1 TS1 mutation G406R, the type-2 Cav1.3 mutation S652L *increases* the sensitivity to the DHP isradipine about 3-fold (Hofer et al., 2020). Therefore, carriers of this mutation should be the most likely candidates for clinical (off-label) testing. The recent observation that nifedipine may improve some neurological symptoms in a patient with a pathogenic Cav1.3 GOF mutation provides some hope for a therapeutic benefit of this strategy (De Mingo Alemany et al., 2020). Evidence for a mutation-specific therapeutic benefit also comes from the observation that some patients with an epileptic encephalopathy due to *CACNA1E* GOF mutations, who were resistant to multiple anti-epileptic drugs, reported significant improvement in seizure control with topiramate. This antiepileptic can inhibit Cav2.3 currents at therapeutically relevant concentrations (Kuzmiski et al., 2005).

Mutation-specific strategies would require new drugs that are capable of reversing some or all of the gating changes of Ca^2+^-channel GOF mutations. As a proof-of-concept it has already been shown that the tert-butyl dihydroquinone (BHQ) can modulate Cav2.1 channels in a manner that oppose altered channel gating of the FHM-1 mutation S218L (type 2, Figures 1D, 4; Inagaki et al., 2014). BHQ inhibited voltage-dependent activation thereby also reducing the enhanced current amplitudes in the mutant channel at negative voltages. At the Drosophila neuromuscular junctions expressing the S218L channel, BHQ largely restored the abnormally elevated evoked postsynaptic potentials and the impaired synaptic plasticity to the wildtype phenotypes.

Another relevant aspect to be considered for GOF mutations is the fact that increased channel activity may not only affect the electrical properties directly by enhancing depolarizing current but may also affect downstream Ca^2+^-dependent signals that could be accessible to therapeutic intervention. Enhanced Ca^2+^-channel signaling may activate Ca^2+^-dependent K^+^-channels, which are closely coupled to different types of Ca^2+^-channels in different cells (Berkefeld et al., 2006; Marcantoni et al., 2010). Another example exists for the TS1 mutation G406R. Roscovitine, a CDK kinase inhibitor, enhances the inactivation of LTCCs and partially reverses the abnormal, slow inactivation of G406R channels (Paşca et al., 2011; Yazawa et al., 2011 for references). It rescues the phenotypes in iPSC-derived cardiomyocytes (prolonged action potential duration) and neurons (aberrant activity-dependent gene-expression) from TS patients (Yazawa et al., 2011; Paşca et al., 2011; Song et al., 2017). In iPSC-derived cardiomyocytes this was explained by enhanced Ca^2+^-dependent activation of the ERK/EGR1 pathway by G406R, which drives overexpression of CDK5. CDK5 inhibition by roscovitine, dominant-negative CDK5 constructs or shRNAs alleviated the TS1 phenotype (Song et al., 2017), and may therefore guide a novel therapeutic approach.

Mutation-specific approaches could also target gating-pore currents. If, as speculated in this review, gating-pore currents contribute to pathology not only in Cav1.1 channelopathies (HypoPP, NormoPP) but also in other Cav channelopathies, then gating-pore blockers would be another attractive approach. Both toxins (e.g., spider toxin Hm-3 from *Heriaeus melloteei*, Männikkö et al., 2018) as well as guanidinium-derivatives (Sokolov et al., 2010) have already been shown to block gating-pore currents through Nav1.4 channel mutants causing periodic paralysis. Together with high-resolution crystal structures of channel mutants (Jiang et al., 2018), molecular dynamics simulations and pharmacophore modeling these studies provided valuable molecular templates for the further development of hit compounds specifically targeting gating-pores.

## Author Contributions

The author confirms being the sole contributor of this work and has approved it for publication.

## Conflict of Interest

The author declares that the research was conducted in the absence of any commercial or financial relationships that could be construed as a potential conflict of interest.

## Footnotes

1 www.guidetopharmacology.org

2 Predicted from truncating mutations within the transmembrane portion of the channel protein (gnomAD Ver2.1.1).
